# Luminescence enhancement by symmetry-breaking in the excited state in radical organic light-emitting diodes

**DOI:** 10.1038/s42004-023-01039-5

**Published:** 2023-11-02

**Authors:** Satoru Ohisa, Satoshi Honda

**Affiliations:** 1 Science & Technology Research Laboratories, Japan Broadcasting Corporation (NHK), 1-10-11 Kinuta, Setagaya-ku, Tokyo 157-8510 Japan; 2 Graduate School of Arts and Sciences, The University of Tokyo, 3-8-1 Komaba, Meguro-ku, Tokyo 153-8902 Japan

**Keywords:** Organic LEDs, Polymers

## Abstract

Organic π-conjugated radicals have recently joined the ranks of high-efficiency light-emitting materials, however, their light-emission mechanism is still a matter of debate. Here, the authors highlight a recently proposed luminescent enhancement mechanism and record-breaking efficiency of a radical organic light-emitting diode.

## Light-emitters for organic light-emitting diodes (OLEDs)

**Fig. 1:**
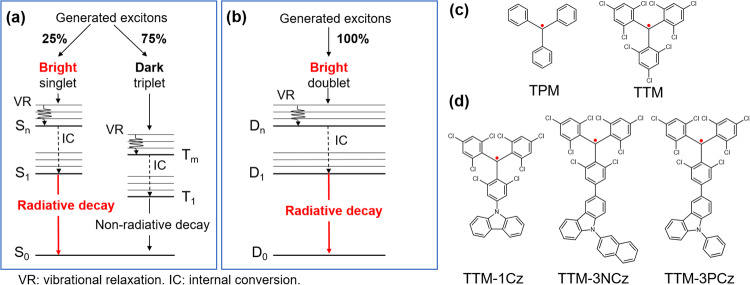
Schematic illustrations of energy level diagrams and organic *π*-conjugated radicals for OLEDs. Differences in emission mechanisms of **a** conventional closed-shell and **b** open-shell systems. Chemical structures of **c** conventional triphenylmethane (TPM) and tris (2,4,6-trichlorophenyl)methane (TTM) radicals and **d** recently developed stable light-emitting radicals for OLEDs operating in the open-shell system.

OLEDs are devices that convert electricity into light and are used as displays and in lighting^1^. Under electric bias, holes and electrons are injected into OLEDs from the anode and cathode. The injected carriers recombine on light-emitting molecules to generate excitons followed by light emission. The luminous efficiency of the OLEDs significantly depends on the type of light emitter used. In the closed-shell light-emitting molecule system (Fig. 1a), the ground state is a singlet, and bright singlet and dark triplet excitons are generated in a 1:3 ratio at carrier recombination. Therefore, the internal quantum efficiency (IQE) is limited to only 25%, as shown in Fig. 1a, corresponding to a limited external quantum efficiency (EQE) of 7.5%, assuming a light out-coupling efficiency (OCE) of 30%. To boost the luminous efficiency in OLEDs, efficient conversion of dark triplet to bright singlet excitons is essential. To realize efficient triplet-singlet conversion, the recent developments of OLEDs have brought various types of light-emitting molecules showing heavy-metal mediated phosphorescence^2^, triplet-triplet annihilation (TTA)^3^ and thermally activated delayed fluorescence^4^, resulting in high-efficiency OLEDs with an EQE of over 30%^5^. On the other hand, recently, open-shell neutral organic π-conjugated radicals (OPRs) have received great attention as a new class of highly efficient light-emitters for OLEDs (Fig. 1b)^6–10^. In this system, both the spin multiplicities of the ground state (*D*_0_) and the lowest-lying excited state (*D*_1_) are doublets, and the *D*_0_–*D*_1_ electronic transition is spin-allowed, making all the generated excitons bright. Therefore, the IQE can reach 100% when OPRs are used as light emitters, as shown in Fig. 1b, and high-efficiency OLEDs can be realized. However, despite these attractive properties of OPRs, they have long not been applied to OLEDs. Significant improvements were necessary in the stability and photoluminescence quantum efficiency (PLQE) for OLED applications, as described below.

## OPRs for OLED application

Organic carbon radicals have lone pairs on carbon atoms and are generally known as unstable chemical species. The insufficient electronegativity of the carbon nucleus cannot bind the lone pair to the carbon atom, and the loosely bound lone pair easily reacts with other chemical species. There are two strategies to stabilize organic carbon radicals (Fig. 1c)^11,12^. One is the delocalization of the lone pair to the π-conjugated system to form OPRs. One representative example is the triphenylmethane (TPM) radical. The lone pair delocalizes to the three phenyl rings and becomes stabilized by π-conjugation. The other is the introduction of steric hindrance near the radical to prevent the chemical reaction. One representative example is the tris(2,4,6-trichlorophenyl)methane (TTM) radical. The substituted chlorine atoms on the phenyl rings act as steric hindrance, and thus, it is more difficult for other molecules to be close to the radical than the unsubstituted TPM radical, bestowing further stabilization to the TTM radical. The TTM radical is stable at room temperature in contact with air and has light-emitting properties, albeit at a low PLQE of several percent. However, the TTM radical is weak against photoexcitation and immediately undergoes decomposition. The recent development of OPRs has overcome these difficulties. The recently developed OPRs are summarized in Fig. 1d. The first TTM radical-based OLED was developed by Peng et al. They utilized a TTM radical substituted by one carbazole (1Cz), developed by Gamero et al., as a light emitter^6, 13^. The TTM-1Cz-based OLED showed deep red electroluminescence with a peak wavelength of 700 nm. However, the achieved maximum EQE was limited to 2.4%. The breakthrough of efficiency improvements was brought by Ai et al.^7^ in 2018. They incorporated 3-substituted-9-phenyl-9H-carbazole (3PCz) and 3-substituted-9-(naphthalen-2-yl)-9H-carbazole (3NCz) into the core TTM radical. TTM-3PCz and TTM-3NCz doped in solid 4,4-bis(carbazol-9-yl)biphenyl (CBP) films (3.0 wt%) exhibited high PLQEs of 60.4% at 695 nm and 85.6% at 707 nm, respectively. TTM-3NCz showed a much higher resistance than TTM in cyclohexane solution under photoexcitation. The PL half-life of TTM was ~20 s, while the PL intensity of TTM-3NCz did not change after several thousand seconds. In addition, TTM-3NCz and TTM-3PCz exhibited high thermal resistance, enabling OLED fabrication using a thermal evaporation process. OLEDs were fabricated using these radicals as light emitters. Surprisingly, a maximum EQE of 17% for TTM-3PCz and 27% for TTM-3NCz were achieved. This high EQE of 27% is close to the theoretical limit of 30%. Thus, Ai et al. proved that the IQE can reach 100% when using radical emitters^7^. This success was achieved by improvements in radical stability and PLQE. The spread of π-conjugation in the substituted TTM is greater than that of TTM, and the lone pair can delocalize more, which is the reason for the stability improvement. In the luminous efficiency, in general, a large overlap between the singly occupied molecular orbital located at the core TTM and the highest occupied molecular orbital located at the carbazole substituent is necessary for a large oscillation strength. In addition, Abdurahman et al. proposed the PLQE enhancement mechanism by intensity borrowing from an intense high-lying electronic transition to a weak low-lying charge-transfer electronic transition^9^. However, the mechanism of luminous efficiency improvement is still a matter of debate.

## Luminous efficiency enhancement by symmetry breaking in the excited state

**Fig. 2:**
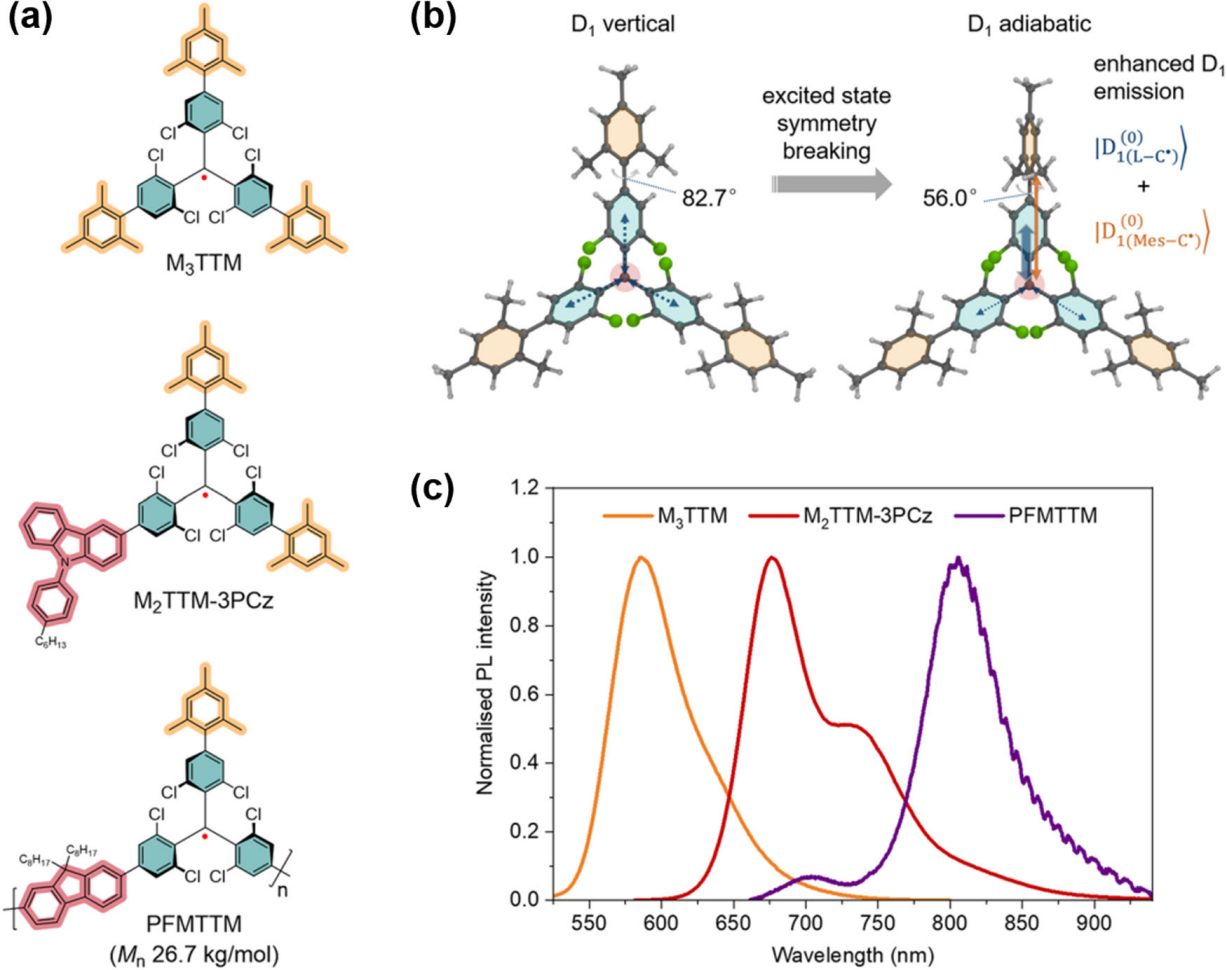
Organic *π*-conjugated radicals improved in luminous efficiency by symmetry breaking and polymerization as tools for shifting luminescent properties. **a** Chemical structures of M_3_TTM, M_2_TTM-3PCz, and PFMTTM radicals. **b** Computationally optimized geometries of the M_3_TTM radical at vertical and adiabatic excited states. D_1_ emission is enhanced by excited-state symmetry breaking. **c** Normalized PL spectra of M_3_TTM, M_2_TTM-3PCz, and PFMTTM. Copyright: Modified from ref. ^10^ used under CC BY.

Very recently, Murto et al. reported the synthesis of mesityl group-substituted TTM radicals (M_1_TTM, M_2_TTM, and M_3_TTM), and M_3_TTM, having the highest symmetry, showed the highest PLQE^10^, which is not explained by the above two mechanisms (Fig. 2a). They explained the mechanism by symmetry breaking in the excited state, where the dihedral angle of one mesitylated phenyl moiety is twisted significantly more than the other two moieties, producing a large transition dipole moment in M_3_TTM and enhancing the PLQE to 28% from 8% of that of the unsubstituted TTM radical in a film doped with CBP host (Fig. 2b). This proposed mechanism is versatile and can broaden the scope of molecular design for PLQE enhancement, and it is worth exploring other substituents to further improve the PLQE. They modified TTM-3PCz with two mesityl substituents (M_2_TTM-3PCz), resulting in an increase in the PLQE to 93% in a film doped with a CBP host. The OLED using M_2_TTM-3PCz was fabricated and showed a record-breaking EQE of 28% at a wavelength of 689 nm. However, efficiency roll-off was observed in the low current density region, and the efficiency decreased at high luminance. In the doped film, time transient PL measurements were performed, and the formation of the intermolecular charge-transfer complex between the luminescent radicals and CBP host was observed, resulting in long-lived excited states on the order of microseconds. In closed-shell systems, TTA, a long-lived triplet-to-triplet reaction, is thought to be responsible for the efficiency roll-off at high current densities^14^. Even in the open-shell system of this study, reactions between doublet and doublet excitons can occur and cause roll-off. The interaction between radicals and host molecules will need to be studied in detail to avoid generating long-lived intermolecular CT states. They also synthesized a main-chain copolymer of mesitylated TTM and 9,9-dioctyl-9H-fluorene (PFMTTM) based on the postpolymerization radicalization of a nonradicalized precursor, which is the first report of a luminescent polymer with radicals embedded in the main-chain. PFMTTM showed significantly redshifted emission beyond 800 nm, which is expected to be a new near-infrared light source (Fig. 2c).

## Outlook

While the present postpolymerization radicalization strategy did not convert all the parent units to their radical forms, as evident from the luminescence from nonradicalized units (Fig. 2c), alternative methodologies for improving the radicalization efficiency or luminescence efficiency, such as polymerization of preradicalized monomers, will lead to the birth of further sophisticated luminescent polymers in combination with the expansion of available monomers and host molecules to replace CBP. Until a decade ago, it was common for radicals not to emit light and could not be applied in OLEDs; however, the recent developments of OPRs overturned this common sense. The future emergence of green- and blue-emitting OPRs with improved stability will open up possibilities for multicolor display applications.
